# Anastomotic leakage following robot-assisted minimally invasive esophagectomy (RAMIE): which anastomosis should be preferred?

**DOI:** 10.1007/s00464-025-11977-x

**Published:** 2025-07-10

**Authors:** Marco Milone, Cezanne D. Kooij, Michele Manigrasso, Lucas Goense, Marc J. van Det, Ewout A. Kouwenhoven, Suzanne S. Gisbertz, Beat P. Müller, Philipp Lingohr, Takeo Fujita, Hans F. Fuchs, Christiane J. Bruns, Dolores T. Krauss, Jan W. Haveman, Boudewijn van Etten, Daniel Perez, Jan-Hendrik Egberts, Paul Turner, Guillaume Piessen, Frank Benedix, Peter P. Grimminger, Luca Bellaio, Vladimir J. Lozanovski, Giovanni Ferrari, Anne Mourregot, Philippe Rouanet, Jens-Peter Hölzen, Mazen A. Juratli, Andreas Pascher, Arul Immanuel, James D. Luketich, Nicholas Baker, Gijs I. van Boxel, Tomas Harustiak, Hecheng Li, Michal Hubka, Zhigang Li, Paolo Strignano, Richard van Hillegersberg, Jelle P. Ruurda, The UGIRA Study Group

**Affiliations:** 1 “Federico II” University of Naples, Naples, Italy; 2 University Medical Center Utrecht, University Utrecht, Heidelberglaan 100, 3584 CX Utrecht, The Netherlands; 3 ZGT Hospital Group Twente, Almelo, The Netherlands; 4 Amsterdam UMC Location University of Amsterdam, Amsterdam, The Netherlands; 5 Cancer Center Amsterdam, Amsterdam, The Netherlands; 6 Department of Surgery, University Digestive Healthcare Center, Basel, Switzerland; 7 University Hospital of Bonn, Bonn, Germany; 8 National Cancer Center Hospital East, Chiba, Japan; 9 University of Cologne, Cologne, Germany; 10 University Medical Center Groningen, University of Groningen, Groningen, The Netherlands; 11 University Hospital Eppendorf, Hamburg, Germany; 12 Israelitisches Krankenhaus Hamburg, Hamburg, Germany; 13 Lancashire Teaching Hospitals NHS Trust, Lancashire, UK; 14 Univ. Lille, CNRS, Inserm, CHU Lille, UMR9020-U1277-CANTHER-Cancer Heterogeneity Plasticity and Resistance to Therapies, Lille, France; 15 University Hospital Magdeburg, Magdeburg, Germany; 16 University Medical Center of the Johannes Gutenberg University, Mainz, Germany; 17 Department of Oncological and Minimally Invasive Surgery Niguarda Hospital, Milan, Italy; 18 Montpellier Cancer Institute, Montpellier, France; 19 Universitätsklinikum Münster, Münster, Germany; 20 Royal Victoria Infirmary Newcastle Upon Tyne, Newcastle Upon Tyne, UK; 21 University Pittsburgh Medical Center, Pittsburgh, PA USA; 22 Portsmouth Hospitals University NHS Trust, Portsmouth, UK; 23 Motol University Hospital, First Faculty of Medicine, Charles University, Prague, Czech Republic; 24 Ruijin Hospital, Shanghai Jiao Tong University School of Medicine, Shanghai, China; 25 Virginia Mason Franciscan Health, Seattle, WA USA; 26 Shanghai Chest Hospital, Shanghai Jiao Tong University School of Medicine, Shanghai, China; 27 Citta’ Della Salute e Della Scienza Turin, Turin, Italy

**Keywords:** Minimally invasive esophagectomy, RAMIE, Anastomotic leakage, Anastomotic technique

## Abstract

**Background:**

The optimal technique for intrathoracic esophagogastric anastomosis in esophagectomy remains undetermined. This study evaluates different anastomotic techniques in robot-assisted minimally invasive esophagectomy (RAMIE) and their impact on anastomotic leakage rates.

**Materials and Methods:**

This observational, retrospective, comparative cohort study analyzed data obtained from the Upper GI International Robotic Association (UGIRA) Esophageal Registry. All consecutive patients with a histologically proven esophageal malignancy who underwent RAMIE with intrathoracic esophagogastrostomy were included. The anastomotic technique was performed based on the clinical judgement and expertise of each individual surgeon. For comparison, the four most common techniques were included: circular end-to-side, linear side-to-side, handsewn end-to-side, and handsewn end-to-end. The primary endpoint of this study was the occurrence of anastomotic leakage, defined by the Esophagectomy Complications Consensus Group as a full-thickness gastrointestinal defect involving the esophagus, anastomosis, staple line, or conduit, regardless of its presentation or method of identification.

**Results:**

Between 2016 and September 2023, 1518 patients were included. Univariable analysis demonstrated that the linear stapled side-to-side anastomosis was associated with the lowest anastomotic leakage rate (14.0%), while the handsewn end-to-end anastomosis had the highest (32.8%) (*p* < 0.001). The anastomotic leakage rates for circular end-to-side and handsewn end-to-side anastomoses were 19.4% and 26.9%, respectively. Multivariable analysis confirmed that anastomotic technique was independently associated with anastomotic leakage. Specifically, handsewn anastomoses were associated with a higher risk of anastomotic leakage for both end-to-side (OR 1.675, 95% CI 1.195–2.348, *p* = 0.003) and end-to-end (OR 2.181, 95% CI 1.403–3.390, *p* < 0.001) techniques compared to circular end-to-side anastomoses.

**Conclusions:**

In RAMIE, linear side-to-side and circular end-to-side stapled anastomoses are associated with lower anastomotic leakage rates compared to handsewn techniques. While acknowledging the multifactorial complexity of anastomotic leakage, these findings favor the use of mechanical stapling in clinical practice.

**Supplementary Information:**

The online version contains supplementary material available at 10.1007/s00464-025-11977-x.

Esophagectomy is the cornerstone of curative treatment for esophageal cancer, typically as part of a multimodal therapy combined with either preoperative chemoradiation or perioperative chemotherapy [1–3]. In recent decades, the adoption of minimally invasive techniques has led to fewer complications and faster recovery compared to open surgery [4, 5]. Additionally, the use of robot-assisted minimally invasive esophagectomy (RAMIE) is increasing globally, with promising outcomes [6, 7]. Despite these advancements, esophagectomy remains a highly invasive procedure associated with a high overall postoperative complication rate of up to 60% [1, 8]. Anastomotic leakage is one of the most severe complications, leading to considerable morbidity, longer hospital stay, increased use of health care resources, and a higher mortality risk [9–11].

Currently, the preferred approach for most surgeons worldwide for mid and distal esophageal tumors is an esophagectomy with an intrathoracic esophagogastric anastomosis [12]. There are several options for creating this esophagogastric anastomosis, largely based on the surgeon’s preference and training [13, 14]. These techniques vary between hand sewn sutures and mechanical stapling, as well as the anatomical configurations used to form the anastomosis (end-to-end, side-to-side, end-to-side) [15]. The objective of the esophagogastric anastomotic technique is to establish a viable, tension-free anastomosis with sufficient clear oncologic margin, irrespective of the method used [14]. Up to date, the optimal technique for esophagectomy with intrathoracic esophagogastric anastomosis for esophageal cancer surgery remained undetermined.

The Upper Gastrointestinal International Robotic Association (UGIRA) established a prospective registry to collect data on robotic surgical techniques and perioperative outcomes from a global cohort of esophageal cancer patients who underwent RAMIE [16]. The primary aim of this study was to compare different esophagogastric anastomotic techniques used in RAMIE with intrathoracic anastomosis within this cohort, with a focus on their impact on anastomotic leakage rates in order to identify a potential superior technique.

## Materials and methods

### Study design

The study is an observational, comparative cohort design, utilizing data obtained from the UGIRA Esophageal Registry. UGIRA was established in 2017 by a leading group of international robotic upper gastrointestinal surgeons and is rapidly expanding, now including 44 centers worldwide that contribute to the UGIRA Esophageal Registry. Its prospectively maintained registry contains data from patients treated with RAMIE in participating UGIRA centers [16]. Institutional review board approval was obtained at the University Medical Center of Utrecht (17/837), and for each participating center, local ethical approval was obtained. Given the anonymous nature of the registry, the requirement for informed consent was waived. The UGIRA Scientific Committee reviewed and approved this research. The paper adheres to the STROBE guidelines for observational studies [17].

### Study population

All consecutive patients with a histologically proven esophageal malignancy who underwent a RAMIE with intrathoracic anastomosis and were registered in the UGIRA Esophageal Registry between 2016 and September 2023 were identified to be included in this multicentric comparative study. Cases involving thoracic phases performed open or with conventional thoracoscopy were excluded. Additionally, patients were excluded if no anastomosis was created, if it involved an esophago-jejunal anastomosis, or it required a colon interposition for esophageal replacement.

### Anastomotic techniques

The anastomotic technique was performed based on the clinical judgement, preference and/or expertise of each individual surgeon. We focused on four anastomotic techniques/combinations: circular end-to-side, linear side-to-side, handsewn end-to-side and handsewn end-to-end. These were selected because they are the most commonly performed anastomotic techniques (in total 96.7%), enabling a meaningful comparison. Other techniques identified in the registry, were excluded from the analysis as they are not typically used or considered feasible in practice.

### Outcomes

The primary endpoint of this study was the occurrence of anastomotic leakage. In the UGIRA Esophageal Registry, complications are registered according to Esophagectomy Complications Consensus Group (ECCG) criteria, which define anastomotic leakage as a full-thickness gastrointestinal defect involving esophagus, anastomosis, staple line, or conduit, irrespective of presentation or method of identification [18]. A comparison among the different types of esophagogastric anastomosis was performed to assess the association between the type of anastomosis and the rate of anastomotic leakage. Furthermore, the study analyzed anastomotic leakage rates over time for each technique to gain insight into the use of specific techniques and their associated leakage rates during the inclusion period. This analysis only includes all full years of the inclusion period (2016–2022) and does not reflect, nor was intended to assess, the impact of surgical experience or learning curves, as centers started registering cases at different points in time.

Additionally, a subgroup analysis was conducted focusing on higher-volume centres. Higher-volume centers were identified as those with a median yearly volume above the third quartile among the participating centers.

The following patients’ and tumors’ characteristics were recorded: gender, age, Body Mass Index (BMI), American Society of Anesthesiologists (ASA) status, tumor’s histology, the adoption of neoadjuvant therapy, clinical T and N-stage.

### Statistical analysis

Statistical analysis was performed using the IBM SPSS Statistics for Windows, Version 29.0 (IBM Corp, Armonk, NY) and R (version 4.0.0 R Project for Statistical Computing). The means ± standard deviation (SD) or medians with interquartile range (IQR) expressed the continuous data, while numbers and percentages the categorical variables. Anastomotic leakage rates were compared using the *χ*^2^ test. Additionally, pairwise comparisons were performed using the *χ*^2^ test with Bonferroni correction. All results are presented as two-tailed values, and the statistical significance was set at *p* < 0.05.

Furthermore, a multivariable analysis, that included all confounding factors (age, Body Mass Index (BMI), American Society of Anesthesiologists (ASA) score, comorbidities, histology, clinical T- and N-stage, neoadjuvant therapy, center volume), was performed to identify the independent association between the different anastomotic techniques with anastomotic leakage. Missing data were considered to be missing at random (number of missing per variable are depicted in Table 1). To address missing data in the variables included in the multivariable analysis, multiple imputation was performed using the iterative Markov chain Monte Carlo method creating 10 datasets [19].

**Table 1:** Patient and tumor characteristics of whole cohort (*n* = 1518) from 27 centers include in the Upper GI International Robotic Association (UGIRA) Esophageal Registry

Variables	RAMIE procedures (*n* = 1518)
Sex	
Male	1242 (81.8%)
Female	275 (18.1%)
Missing	1 (0.1%)
Age, years (median [IQR] Missing	66 [59–72] 1 (0.1%)
BMI, kg/m^2^ (mean [SD]) Missing	26.3 (± 4.6) 42 (2.8%)
ASA score	
1	100 (6.6%)
2	772 (50.9%)
3	580 (38.2%)
4	24 (15.8%)
Missing	42 (2.8%)
Any comorbidity	1138 (75.0%)
Pulmonary comorbidity	228 (15.0%)
Cardiac comorbidity	493 (32.5%)
Vascular comorbidity	406 (26.7%)
Oncological comorbidity	151 (9.9%)
Neurological comorbidity	89 (5.9%)
Diabetes	234 (15.4%)
Histology	
Adenocarcinoma	1270 (83.7%)
Squamous cell carcinoma	248 (16.3%)
Neoadjuvant therapy	1249 (85.5%)
Chemoradiotherapy	827 (54.5%)
Chemotherapy alone	450 (29.6%)
Radiotherapy alone	3 (0.2%)
Other	17 (1.1%)
Missing	4 (0.3%)
Clinical T-stage	
T1	120 (7.9%)
T2	276 (18.2%)
T3	1037 (68.3%)
T4	53 (3.5%)
Missing	32 (2.1%)
Clinical N-stage	
N0	510 (33.6%)
N1	759 (50.0%)
N2	193 (12.7%)
N3	31 (2.0%)
Missing	25 (1.6%)

*BMI* Body Mass Index (kg/m^2^), *ASA* American Society of Anesthesiologists, *SD* standard deviation, *IQR* interquartile range

## Results

### Patients characteristics

**Fig. 1:**
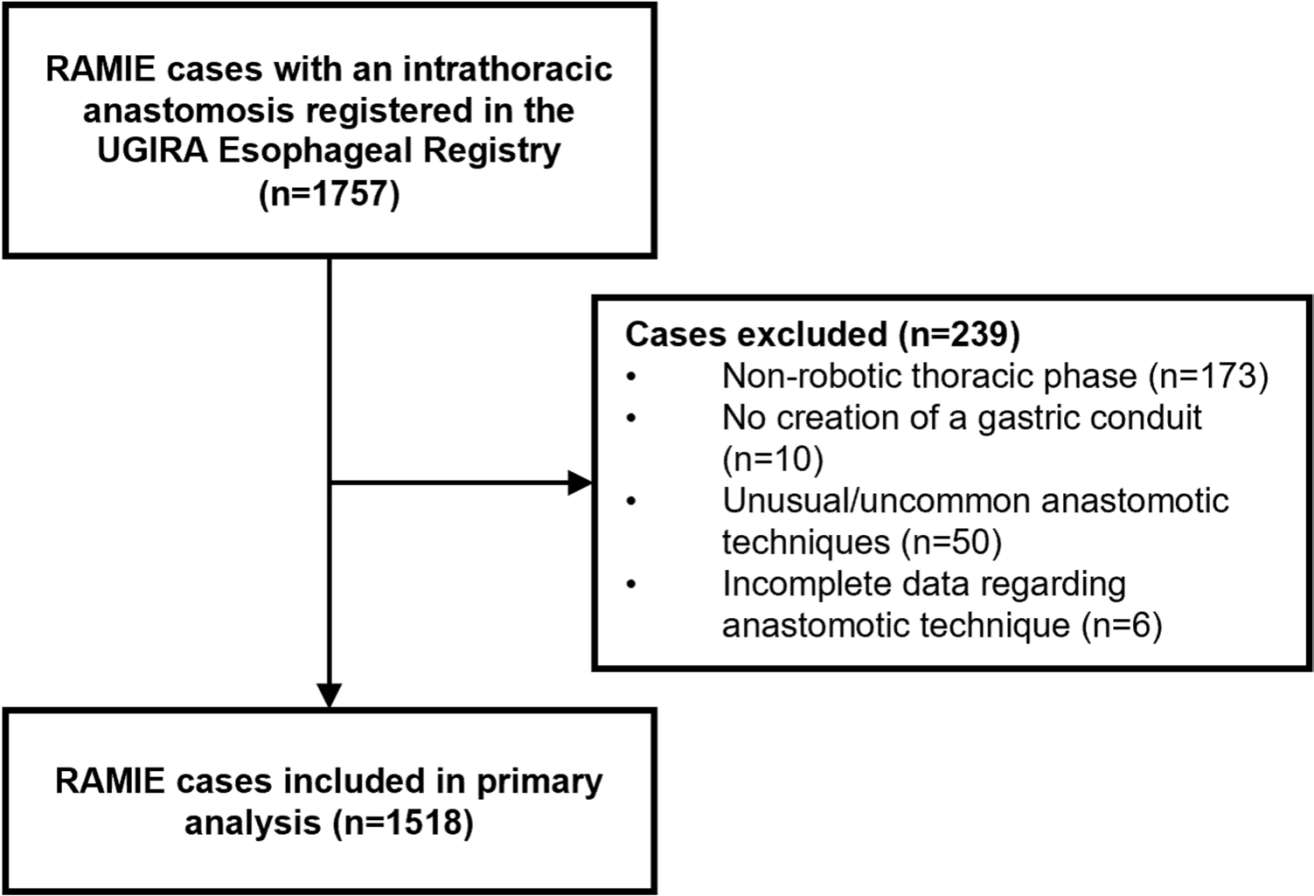
Flowchart of patient inclusion

As demonstrated in Fig. 1, 1757 patients were treated for esophageal cancer and registered in the UGIRA Esophageal Registry by 29 centers. After excluding cases based on a non-robotic thoracic phase (*n* = 173), no creation of a gastric conduit (*n* = 10), performance of an unusual/uncommon anastomotic technique (*n* = 50) or incomplete data regarding anastomotic technique (*n* = 6), 1518 RAMIE procedures performed by 27 centers were included. Among these centers, 21 used circular end-to-side stapling, 8 used linear end-to-side stapling, 9 used handsewn end-to-side, and 8 used handsewn end-to-end techniques. Additionally, of the 27 centers, 14 used one anastomotic technique, 7 used two techniques, and 6 used three techniques during the study period.

Patient and tumor characteristics are reported in Table 1. Of the included patients, 1242 (81.8%) were male, with a median age of 66 years [IQR 59–72] and a mean BMI of 26.3 kg/m^2^ (SD ± 4.6). The majority of patients had an ASA score of 2 (*n* = 772, 50.9%) or 3 (*n* = 580, 38.2%), and 75% of patients had a comorbidity. The majority of patients (*n* = 1270, 83.7%) had an adenocarcinoma. Most patients (*n* = 1249, 85.5%) received neoadjuvant therapy; 827 patients (54.5%) were treated with chemoradiotherapy, 450 (29.6%) with chemotherapy alone, 3 (0.2%) with radiotherapy alone and 17 (1.1%) with another type of neoadjuvant therapy. The majority of patients had a cT3-stage (*n* = 1037, 68.3%) and either cN0-stage (*n* = 510, 33.6%) or cN1-stage (*n* = 759, 50%).

### Outcome analysis

Table 2 presents the total number of RAMIE procedures for each esophagogastric anastomotic technique. The univariable analysis demonstrated that linear stapled side-to-side and circular stapled end-to-side anastomoses were associated with the lowest rates (14.0% and 19.4%, respectively) and the handsewn end-to-side and end-to-end anastomoses with the highest rates (26.9% and 32.8%, respectively) of anastomotic leakage (*p* < 0.001). Pairwise comparisons showed that circular stapled end-to-side anastomoses exhibited significantly lower rates of anastomotic leakage in comparison to handsewn end-to-end anastomoses (*p* < 0.001). Furthermore, linear stapled side-to-side anastomoses demonstrated significantly lower anastomotic leakage rates than both handsewn end-to-side (*p* < 0.001) and end-to-end anastomoses (*p* < 0.001). No significant difference in leakage rates was observed between the linear stapled side-to-side and circular stapled end-to-side anastomosis (*p* = 0.270).

**Table 2:** Anastomotic leakage after esophagogastric anastomosis

Esophagogastric anastomosis	Total no of patients	Leakage (%)	*p*-value^^^
Esophagogastric anastomosis			**< 0.001**
Circular end-to-side	882	171 (19.4%)	
Linear side-to-side	271	38 (14.0%)	
Handsewn end-to-side	249	67 (26.9%)	
Handsewn end-to-end	116	38 (32.8%)	
Pairwise comparisons			
Circular end-to-side versus linear side-to-side		19.4%/14.0%	0.270
Circular end-to-side versus handsewn end-to-side		19.4%/26.9%	0.060
Circular end-to-side versus handsewn end-to-end		19.4%/32.8%	**< 0.001**
Linear side-to-side versus handsewn end-to-side		14.0%/26.9%	**< 0.001**
Linear side-to-side versus handsewn end-to-end		14.0%/32.8%	**< 0.001**
Handsewn end-to-side versus handsewn end-to-end		26.9%/32.8%	1.000

^^^ Calculated with the *χ*^2^ test, and in pairwise comparisons adjusted for multiple comparisons using the Bonferroni correction

Bold value indicates statistical significance (*p* < 0.05)

Supplementary Figure 1 illustrates the anastomotic leakage rates for each anastomotic technique over the inclusion period. Circular end-to-side stapling showed relatively stable leakage rates throughout the study period. Linear side-to-side stapling was first registered in 2018, with leakage rates in the last 2 years of the study period being relatively low (10.2% and 5.4%). Handsewn end-to-side anastomoses had leakage rates just above 30% in the early years of registration, and a rate of 22.9% in the last year. Handsewn end-to-end anastomoses exhibited the highest leakage rates over time, although group sizes were relatively small compared to the other anastomotic groups.

### Subgroup analyses

**Fig. 2:**
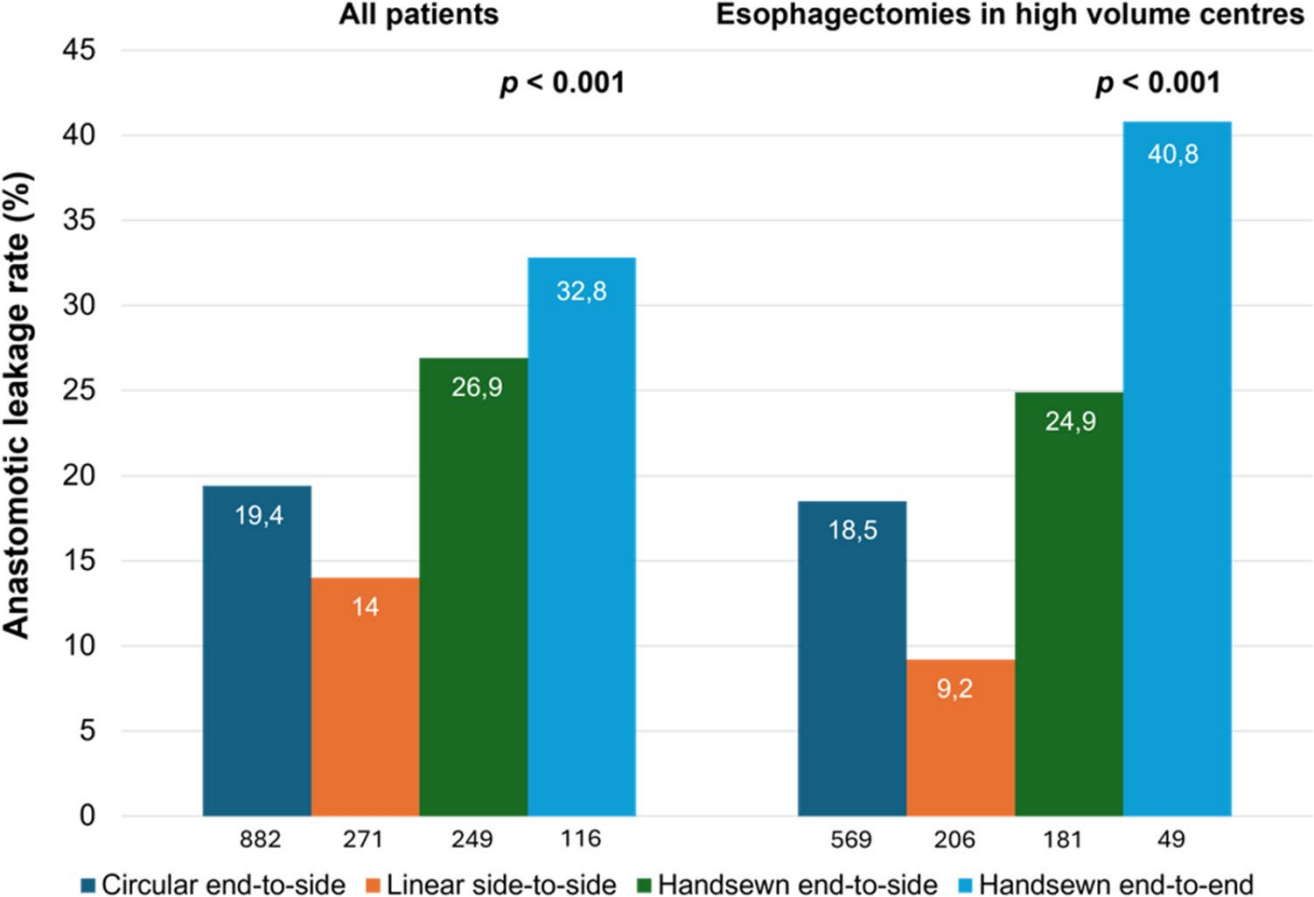
Comparison of anastomotic leakage rates in RAMIE cases with different anastomotic techniques. All included cases (left); Cases performed in high volume centers (20 or more cases/year) (right)

The univariable subgroup analysis focused on RAMIE procedures performed in higher-volume centers, which were defined as those with a median yearly volume above the third quartile among the participating centers. The third quartile for center volume was 19.5, so centers included in the subgroup analysis had to perform 20 or more cases per year. This analysis included 1005 esophagectomies from 7 centers. The subgroup analysis demonstrated similar results to the primary analysis, as depicted in Fig. 2. The linear stapled side-to-side anastomosis was associated with the lowest rate (9.2%) and the handsewn end-to-end anastomosis with the highest rate (40.8%) of anastomotic leakage (*p* < 0.001).

### Multivariable analysis

Multivariable analysis demonstrated that the type of anastomosis was independently associated with anastomotic leakage (Table 3). Specifically, handsewn anastomoses were associated with a higher risk of anastomotic leakage for both end-to-side (OR 1.675, 95% CI 1.195–2.348, *p* = 0.003) and end-to-end (OR 2.181, 95% CI 1.403–3.390, *p* < 0.001) techniques compared to circular end-to-side anastomoses.

**Table 3:** Multivariable analysis (logistic regression) on the impact of esophagogastric anastomosis on anastomotic leakage

Characteristics	Variable	OR (95%CI)	*p*-value^*^
Age	Continuous	1.009 (0.995,1.023)	0.229
BMI	Continuous	1.016 (0.988,1.045)	0.275
ASA Score	1–2 versus 3–4	1.183 (0.907,1.543)	0.215
Histology	AC versus SCC	0.607 (0.410,0.897)	**0.012**
Neoadjuvant therapy	No versus yes	0.927 (0.610,1.411)	0.725
cT	cT1-2 versus cT3-4	0.800 (0.577,1.108)	0.179
cN	cN0 versus cN +	1.461 (1.078,1.980)	**0.015**
Center volume	Low versus high (Q3)	0.758 (0.580,0.991)	**0.042**
Esophagogastric anastomosis			
Circular end-to-side (ref.)		–	–
Linear side-to-side		0.197 (0.472,1.029)	0.069
Handsewn end-to-side		1.675 (1.195,2.348)	**0.003**
Handsewn end-to-end		2.181 (1.403,3.390)	**< 0.001**

*OR* Odds ratio, *CI* Confidence interval

^*^ The *p*-values presented are pooled from all (*n* = 10) imputed datasets

Bold value indicates statistical significance (*p* < 0.05)

## Discussion

This study assessed the most commonly used techniques for the intrathoracic esophagogastric anastomosis in RAMIE. Linear side-to-side stapling and circular end-to-side stapling were associated with lower rates of anastomotic leakage, at 14.0% and 19.4%, respectively. In contrast, handsewn suturing demonstrated higher leakage rates, with end-to-side anastomosis at 26.9% and end-to-end anastomosis at 32.8%. Additionally, the multivariable analysis confirmed an independent association between the applied technique and the occurrence of anastomotic leakage, with handsewn sutured anastomoses significantly increasing this risk.

The handsewn method was the first type of anastomosis for esophageal reconstruction, offering satisfactory outcomes at lower costs but requiring significant skill and time [20]. While some surgeons still consider the handsewn technique more reliable, the advent of mechanical stapling revolutionized the field, introducing benefits such as shorter operative times, procedural uniformity, and consistent anastomotic integrity, with titanium staples minimizing tissue reaction [20]. However, stapled techniques are more expensive, and while an association with a higher likelihood of strictures has been suggested, this remains inconclusive and is primarily based on comparisons between circular stapling and handsewn techniques [20, 21]. Circular staplers simplify the procedure by leaving no common opening for the anastomosis, with a predetermined aperture size. In contrast, linear staplers require closure of a common opening but allow for greater variability in aperture size [20]. Additionally, the side-to-side orientation of linear staplers may reduce traction-related leakage [22].

Anastomotic leakage rates following esophagectomy range from 5 to 40%, with lower rates reported for intrathoracic compared to cervical anastomoses [9, 23–25]. Despite the influence of anastomotic techniques, alongside patient and perioperative factors, no consensus exists on the optimal methods to minimize leakage [9, 25]. Previous meta-analyses of randomized controlled trials (RCTs) present mixed findings regarding anastomotic techniques. One found no significant difference in leakage rates between handsewn and circular stapled anastomoses [26], while another reported lower leakage rates with linear stapling compared to handsewn techniques [27]. Additionally, one meta-analysis suggested that stapled anastomoses generally result in lower leakage rates than handsewn ones [28]. Comparisons between linear and circular stapling showed no significant difference in leakage rates [29], but a network meta-analysis identified linear stapling as superior in reducing leakage rates compared to other methods [22]. This study aligns with the broader literature, suggesting that stapling techniques, particularly linear stapling, may offer advantages in reducing anastomotic leakage. However, the multifactorial and complex etiology of anastomotic leakage necessitates cautious interpretation of these findings.

Several limitations of this study should be acknowledged. First, the registry lacks data on the rationale behind a surgeon’s choice of a particular anastomotic technique, limiting insight into the decision-making process. Furthermore, due to the involvement of multiple surgeons at some centers and the anonymity of the registry, it is not possible to determine whether the choice of technique was surgeon-specific or random. Second, while the heterogeneity of the global cohort of patients treated with RAMIE allows for comparison of different anastomotic techniques, it also introduces variability in clinical practices, such as perioperative care protocols, as well as the impact of the learning curve and surgical experience—details that could not be retrieved from the registry. Third, key technical aspects—such as pleural flaps, omental wraps, and tension releasing sutures—were not captured in the registry, yet these factors may influence anastomotic outcomes. Additionally, the severity of anastomotic leakage was not included as a variable in the registry, which is essential for understanding and interpreting its clinical implications. Finally, there was no information on long-term outcomes, such as stenosis.

Nonetheless, this study is valuable for its focus exclusively on RAMIE procedures with intrathoracic anastomosis. Additionally, it includes a large global cohort from 27 centers, enabling robust multivariable analysis. Furthermore, the subgroup analysis provided insights into the impact of surgeon experience and center volume, demonstrating lower leakage rates across all groups except for the relatively small group of handsewn end-to-end anastomoses. In this subgroup, the leakage rate was higher in higher-volume centers, but the small sample size makes interpretation challenging.

The UGIRA registry is a unique international collaboration among centers performing robot-assisted upper GI surgery. The registry offers valuable research opportunities and the potential to enhance the overall outcomes of robotic surgery by identifying effective techniques and centers with optimal results. This valuable information can facilitate peer to peer visits, fellowships, training courses and presentations at scientific meetings.

In conclusion, this study demonstrated an association between the techniques employed for intrathoracic esophagogastric anastomosis in RAMIE and the occurrence of anastomotic leakage. Side-to-side linear and end-to-side circular stapling resulted in better outcomes than end-to-side and end-to-end handsewn suturing. Despite the multifactorial nature of anastomotic leakage, these results provide guidance for clinical practice, favoring mechanical stapling.

## Supplementary Information

Below is the link to the electronic supplementary material.

Supplementary file1 (DOCX 114 KB)

## References

[1] Low DE et al (2019) Benchmarking complications associated with esophagectomy. Ann Surg 269(2):291–298. 10.1097/SLA.0000000000002611 29206677 10.1097/SLA.0000000000002611

[2] Kelly RJ (2019) Emerging multimodality approaches to treat localized esophageal cancer. J Natl Compr Canc Netw 17(8):1009–1014. 10.6004/jnccn.2019.7337 31390584 10.6004/jnccn.2019.7337

[3] Harada K, Rogers JE, Iwatsuki M, Yamashita K, Baba H, Ajani JA (2020) Recent advances in treating oesophageal cancer. F1000Res 9:1189. 10.12688/f1000research.22926.1 10.12688/f1000research.22926.1 PMC7531047 33042518

[4] Yibulayin W, Abulizi S, Lv H, Sun W (2016) Minimally invasive oesophagectomy versus open esophagectomy for resectable esophageal cancer: a meta-analysis. World J Surg Oncol 14(1):304. 10.1186/s12957-016-1062-7 27927246 10.1186/s12957-016-1062-7 PMC5143462

[5] Luketich JD et al (2012) Outcomes after minimally invasive esophagectomy. Ann Surg 256(1):95–103. 10.1097/SLA.0b013e3182590603 22668811 10.1097/SLA.0b013e3182590603 PMC4103614

[6] van der Sluis PC et al (2019) Robot-assisted minimally invasive thoracolaparoscopic esophagectomy versus open transthoracic esophagectomy for resectable esophageal cancer. Ann Surg 269(4):621–630. 10.1097/SLA.0000000000003031 30308612 10.1097/SLA.0000000000003031

[7] Zhang Y et al (2023) Robotic versus conventional minimally invasive esophagectomy for esophageal cancer. Ann Surg 278(1):39–50. 10.1097/SLA.0000000000005782 36538615 10.1097/SLA.0000000000005782

[8] Bras Harriott C, Angeramo CA, Casas MA, Schlottmann F (2022) Open versus hybrid versus totally minimally invasive Ivor Lewis esophagectomy: systematic review and meta-analysis. J Thorac Cardiovasc Surg 164(6):e233–e254. 10.1016/j.jtcvs.2021.12.051 35164948 10.1016/j.jtcvs.2021.12.051

[9] Kassis ES, Kosinski AS, Ross P, Koppes KE, Donahue JM, Daniel VC (2013) Predictors of anastomotic leak after esophagectomy: an analysis of the society of thoracic surgeons general thoracic database. Ann Thorac Surg 96(6):1919–1926. 10.1016/j.athoracsur.2013.07.119 24075499 10.1016/j.athoracsur.2013.07.119

[10] Goense L, Meziani J, Ruurda JP, van Hillegersberg R (2019) Impact of postoperative complications on outcomes after oesophagectomy for cancer. J Br Surg 106(1):111–119. 10.1002/bjs.11000 10.1002/bjs.11000 30370938

[11] Goense L, van Dijk WA, Govaert JA, van Rossum PSN, Ruurda JP, van Hillegersberg R (2017) Hospital costs of complications after esophagectomy for cancer. Eur J Surg Oncol EJSO 43(4):696–702. 10.1016/j.ejso.2016.11.013 28012715 10.1016/j.ejso.2016.11.013

[12] de Groot EM, Goense L, Kingma L, Haverkamp L, Ruurda JP, van Hillegersberg R (2023) Trends in surgical techniques for the treatment of esophageal and gastroesophageal junction cancer: the 2022 update. Dis Esophagus. 10.1093/dote/doac099 36636763 10.1093/dote/doac099 PMC10317002

[13] Chen B, Xia P, Tang W, Huang S (2023) Which anastomotic techniques is the best choice for cervical esophagogastric anastomosis in esophagectomy? A Bayesian network meta-analysis. J Gastrointest Surg 27(2):422–432. 10.1007/s11605-022-05482-y 36417036 10.1007/s11605-022-05482-y

[14] Herron R, Abbas G (2021) Techniques of esophageal anastomoses for esophagectomy. Surg Clin North Am 101(3):511–524. 10.1016/j.suc.2021.03.012 34048770 10.1016/j.suc.2021.03.012

[15] Carr RA, Molena D (2021) Minimally invasive esophagectomy: anastomotic techniques. Ann Esophagus 4:19–19. 10.21037/aoe-20-40

[16] Kingma BF et al (2022) Worldwide techniques and outcomes in robot-assisted minimally invasive esophagectomy (RAMIE). Ann Surg 276(5):e386–e392. 10.1097/SLA.0000000000004550 33177354 10.1097/SLA.0000000000004550

[17] Cuschieri S (2019) The STROBE guidelines. Saudi J Anaesth 13(5):31. 10.4103/sja.SJA_543_18 10.4103/sja.SJA_543_18 PMC6398292 30930717

[18] Low DE et al (2015) International consensus on standardization of data collection for complications associated with esophagectomy. Ann Surg 262(2):286–294. 10.1097/SLA.0000000000001098 25607756 10.1097/SLA.0000000000001098

[19] Eekhout I, de Vet HC, de Boer MR, Twisk JW, Heymans MW (2018) Passive imputation and parcel summaries are both valid to handle missing items in studies with many multi-item scales. Stat Methods Med Res 27(4):1128–1140. 10.1177/0962280216654511 27334917 10.1177/0962280216654511

[20] Yuan Y, Wang K-N, Chen L-Q (2015) Esophageal anastomosis. Dis Esophagus 28(2):127–137. 10.1111/dote.12171 24438553 10.1111/dote.12171

[21] Markar SR, Karthikesalingam A, Vyas S, Hashemi M, Winslet M (2011) Hand-Sewn versus stapled oesophago-gastric anastomosis: systematic review and meta-analysis. J Gastrointest Surg 15(5):876–884. 10.1007/s11605-011-1426-9 21271360 10.1007/s11605-011-1426-9

[22] Kamarajah SK, Bundred JR, Singh P, Pasquali S, Griffiths EA (2020) Anastomotic techniques for oesophagectomy for malignancy: systematic review and network meta-analysis. BJS Open 4(4):563–576. 10.1002/bjs5.50298 32445431 10.1002/bjs5.50298 PMC7397345

[23] Low DE (2011) Diagnosis and management of anastomotic leaks after esophagectomy. J Gastrointest Surg 15(8):1319–1322. 10.1007/s11605-011-1511-0 21557015 10.1007/s11605-011-1511-0

[24] Biere SSAY, Maas KW, Cuesta MA, van der Peet DL (2011) Cervical or thoracic anastomosis after esophagectomy for cancer: a systematic review and meta-analysis. Dig Surg 28(1):29–35. 10.1159/000322014 21293129 10.1159/000322014

[25] Gooszen JAH, Goense L, Gisbertz SS, Ruurda JP, van Hillegersberg R, van Berge Henegouwen MI (2018) Intrathoracic *versus* cervical anastomosis and predictors of anastomotic leakage after oesophagectomy for cancer. Br J Surg 105(5):552–560. 10.1002/bjs.10728 29412450 10.1002/bjs.10728 PMC5900725

[26] Honda M, Kuriyama A, Noma H, Nunobe S, Furukawa TA (2013) Hand-sewn versus mechanical esophagogastric anastomosis after esophagectomy. Ann Surg 257(2):238–248. 10.1097/SLA.0b013e31826d4723 23001084 10.1097/SLA.0b013e31826d4723

[27] Deng X-F, Liu Q-X, Zhou D, Min J-X, Dai J-G (2015) Hand-sewn *vs* linearly stapled esophagogastric anastomosis for esophageal cancer: a meta-analysis. World J Gastroenterol 21(15):4757–4764. 10.3748/wjg.v21.i15.4757 25914488 10.3748/wjg.v21.i15.4757 PMC4402326

[28] Järvinen T, Cools-Lartigue J, Robinson E, Räsänen J, Ilonen I (2021) Hand-sewn versus stapled anastomoses for esophagectomy: we will probably never know which is better. JTCVS Open 7:338–352. 10.1016/j.xjon.2021.07.021 36003702 10.1016/j.xjon.2021.07.021 PMC9390502

[29] Zhou D, Liu Q-X, Deng X-F, Min J-X, Dai J-G (2015) Comparison of two different mechanical esophagogastric anastomosis in esophageal cancer patients: a meta-analysis. J Cardiothorac Surg 10(1):67. 10.1186/s13019-015-0271-4 25952323 10.1186/s13019-015-0271-4 PMC4456702

