# Supplementary material for: Anastomotic leakage following robot-assisted minimally invasive esophagectomy (RAMIE): which anastomosis should be preferred?
Source: Surg Endosc. 2025 Jul 10;39(9):5604–12. doi: 10.1007/s00464-025-11977-x (PMC12408781; doi:10.1007/s00464-025-11977-x)
Supplement: Supplementary file 1 — Supplementary file1 (DOCX 114 KB) [file 464_2025_11977_MOESM1_ESM.docx]

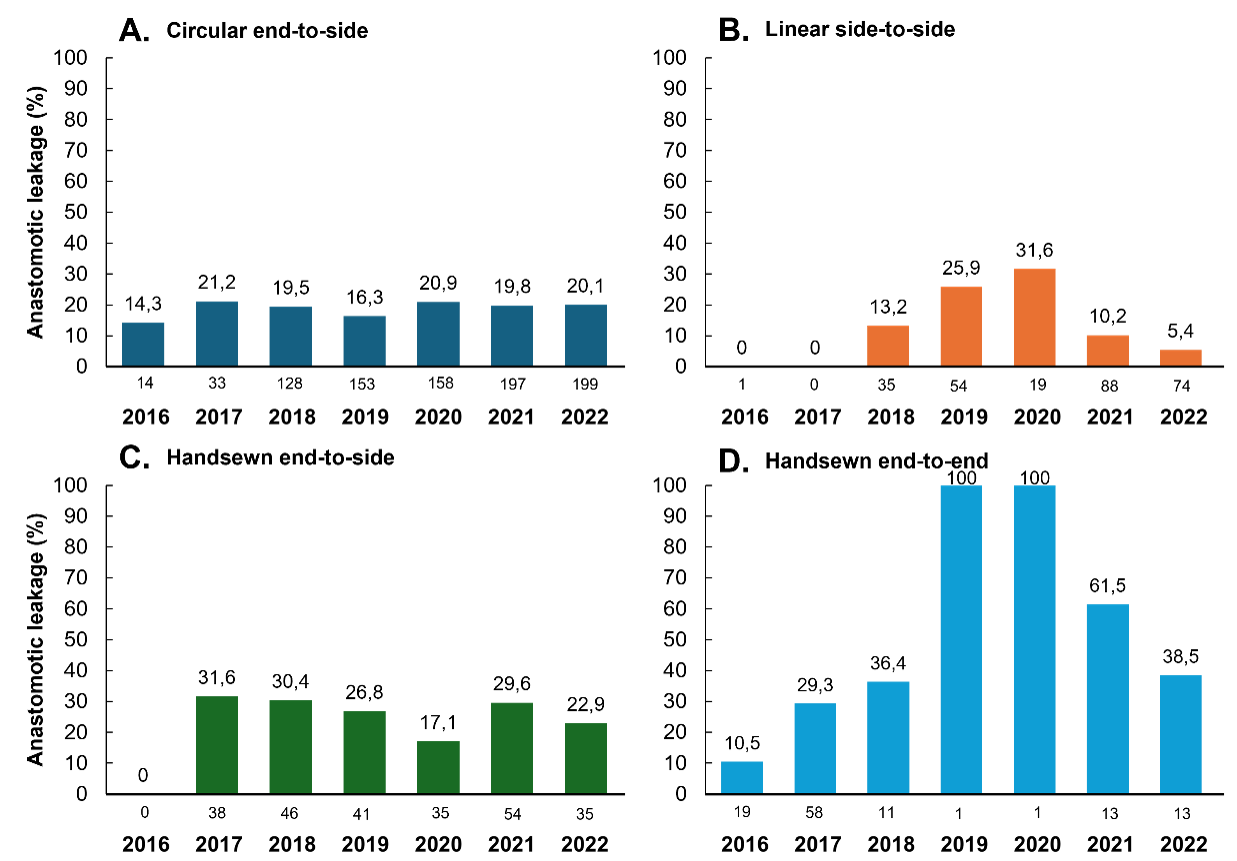
**eFigure 1:** Anastomotic leakage rates over time per anastomotic technique (A. Ciruclar end-to-side; B. Linear side-to-side; C. Handsewn end-to-side; D: Handsewn end-to-end)
